# The Role of Orthoptists in Refraction

**DOI:** 10.22599/bioj.384

**Published:** 2025-02-14

**Authors:** Miriam L. Conway, Rakhee Shah, Elizabeth Chapman, Bruce J. W. Evans

**Affiliations:** 1 City St George’s, University of London, UK; 2 HOYA Vision Care, Amsterdam, the Netherlands; 3 Fellowship Diploma of the Association of British Dispensing Opticans, UK

**Keywords:** orthoptist, refraction, retinoscopy, Opticians Act

## Abstract

**Purpose:**

In 2022, the General Optical Council initiated a call for evidence concerning the Opticians Act. This consultation aimed to gather input and evidence relevant to potential modifications to the Opticians Act. One piece of research that was commissioned aimed to investigate the role of orthoptists in refraction.

**Method:**

We invited a range of eye care practitioners to participate in an online virtual focus group. Focus group discussions were audio-recorded, transcribed, and thematically analysed.

**Results:**

Two focus group discussions involving sixteen eye care professionals were completed. Findings confirm some orthoptists are already performing refraction tasks within the hospital eye service, primarily with young children using cycloplegic retinoscopy. Participants indicated that, at present, orthoptists refract on behalf of ophthalmologists who issue spectacle prescriptions based on the findings of the orthoptist’s refraction. Potential benefits of orthoptists undertaking refraction responsibilities were discussed including the ability to conduct retinoscopy in hospital paediatric clinics where services are in high demand and there is often a shortage of refraction appointments. This shift could lead to decreased NHS waiting times, fewer patient appointments, improved clinical decision-making, and facilitate the assessment of accommodative dysfunction. Overall, the group were positive towards orthoptists conducting refractions and issuing optical prescriptions but with specific conditions: limited to hospital settings, necessitating adequate postgraduate training, supervision by a hospital eye service consultant, and regular ocular health assessments.

**Conclusion:**

This study assessed the involvement of orthoptists in present and prospective refraction services, including their potential to legally issue optical prescriptions. The research outlined both the potential benefits and mitigating strategies to address concerns if orthoptists could issue optical prescriptions.

## Introduction

Clinically, the term refraction is defined as the process of measuring and correcting the refractive error of the eye (Millodot, 2017). Refraction is a crucial component of the eye examination to assess vision and detect potential eye health issues. Objective refraction, not requiring patient response, can be performed using an autorefractor or retinoscope. The main method of retinoscopy, distance fixation, can be undertaken in eyes that are in their natural state or when the eye’s ability to accommodate has been temporarily paralysed using cycloplegic refraction. Mohindra retinoscopy is a technique that allows a reasonable estimate of the cycloplegic refractive error without using cycloplegic agent (Mohindra, 1975). For pre-presbyopic patients when cycloplegia is not used, additional retinoscopy techniques are available to assess accommodative function. These are referred to as dynamic retinoscopy and include monocular estimate method (MEM) retinoscopy and Nott retinoscopy (Rabbetts, 2007).

In patients who can communicate and co-operate, objective refraction typically is followed by subjective refraction, where patients provide feedback to fine-tune the prescription. Retinoscopy is particularly valuable in children and individuals with communication challenges or those where a previously determined refractive error is not available as a starting point for refraction. Mastering retinoscopy requires extensive training and practice but offers unique benefits not always achievable through other testing methods like autorefractors.

In optometry degree programmes, retinoscopy training begins in the first year typically with around ten hours of supervised practice, assessed towards year-end. In the second year, approximately 5–10 hours are dedicated to retinoscopy as part of full refraction training; however, this may vary in different optometry schools. Before September 2023, all stage one optometry students were required to complete a minimum of 18 safe refractions in primary care clinics (GOC, 2015), with additional retinoscopy experience in other clinics. Under these training requirements pre-registration optometrists were expected to perform a minimum of 350 refractions, with competency assessments, including retinoscopy, throughout the second, third, and pre-registration years (GOC, 2015). The time allocated for conducting retinoscopy specifically on paediatric patients and those with learning disabilities was generally limited and not clearly quantified. It is important to highlight that universities are now transitioning to the updated education and training requirements (ETR), which has moved away from a numerical competency-based approach towards a new outcomes-based approach (GOC, 2021). Most students who began their studies in 2024 are enroled in adapted courses, rendering the 2015 handbooks inapplicable.

Orthoptists possess specialised skills in conducting eye tests for younger paediatric patients and individuals with learning disabilities due to significant hours spent training in this area. Contemporary training for orthoptists includes refraction (HCPC, 2023). The British and Irish Orthoptic Society (BIOS) and the Royal College of Ophthalmologists (RCOpth) recently conducted a mapping exercise. Orthoptic education was mapped to the RCOpth curriculum confirming that the training provided to trainee ophthalmologists regarding refraction aligns with that imparted to orthoptists in terms of knowledge and skills (BIOS, 2024). The British and Irish Orthoptic Society also recognise that many orthoptists routinely perform safe refractive assessments as part of their clinical evaluations. However, they are not legally allowed to issue prescriptions, which can lead to delays and the need for additional visits with another eye care professional. To address these legislative constraints, they follow local protocols for issuing Hospital Eye Service (HES) prescriptions (BIOS, 2024).

The General Optical Council (GOC) is the regulatory body for the optical professions in the UK, and regulates around 34,000 optometrists, dispensing opticians, student optometrists, student dispensing opticians, and optical businesses. In the UK, the current model of sight testing includes both refraction and eye health checks. Section 24 of the Opticians Act 1989 (*The Opticians Act*, 1989) (‘the Act’) stipulates that a sight test can be conducted only by an optometrist or a registered medical practitioner (with special provision for students under supervision). Section 27(7) of the Act and Article 3(2)(a) of the Sale of Optical Appliances Order 1984 states that spectacles prescriptions can be issued only by an optometrist or registered medical practitioner.

In 2013, the GOC stated that no part of the sight test can be delegated, even under the supervision of an optometrist or registered medical practitioner (GOC, 2013). In 2022, the GOC evaluated the possibility of revising its stance on delegating refraction, considering advancements in technology and in the profession. The consideration involved potentially permitting refraction delegation under the supervision of either an optometrist or a registered medical practitioner. To inform this consideration, the GOC commissioned research on several topics (GOC, 2022). In relation to orthoptists, they stated they would like research to help them understand ‘more about the role of orthoptists in refraction and sight testing – this should include their role in hospitals and vision screening, and their experience in undertaking refraction.’ The authors investigated and published a report on the topic titled ‘Clinical advice on refraction for sight testing’ (Evans *et al*., 2023). The subject matter was examined through three studies: a survey on sight test delivery, a Delphi study assessing the potential impact of separating sight test components and focus groups investigating the role of orthoptists in refraction and sight testing. The primary aim of the third study, reported in this research paper, was to use focus groups to explore the role of orthoptists in refraction and sight testing.

## Methods

Focus groups were chosen due to their ability to provide an in-depth examination of participants’ experiences, attitudes, and opinions in a group setting. This setting encourages interaction, often uncovering insights that may not surface in surveys or one-on-one interviews. Additionally, facilitators can clarify responses and explore specific topics in greater depth, more effectively than in individual interviews. Ethical approval for all three studies was granted by the Institute of Optometry, ethics committee (reference 22/BE/0101). All procedures in this study were in accordance with the tenets of the Declaration of Helsinki. A signed declaration of informed consent was obtained from all participants. The study utilised purposive sampling (Etikan, 2016), to select experienced participants who were able to contribute to the research objective of understanding the role of orthoptists in refraction and sight testing. This type of sampling is essential when the research requires detailed, context-specific, and in-depth understanding from a select group of knowledgeable participants. To achieve this, we identified orthoptists with varying levels of training and experience in refraction across the UK. We also included student orthoptists to evaluate current training, inviting participants from all four universities offering undergraduate orthoptics courses. Additionally, we invited optometrists who had collaborated with orthoptists in hospital or community settings and dual-registered (orthoptist and optometrist) clinicians to provide unique insights into both roles. Some participants were familiar to the main author (MC), while others were recommended by colleagues following research and email outreach to various hospitals, universities, and contacts. Two participants were known to the facilitator. All parties were contacted via email.

Two focus group discussions were conducted between November 2022 and December 2022. The duration of each focus group was less than 60 minutes. Seven participants attended focus group one and nine attended focus group two. The demographic details of the participants in the two groups are given in Tables 1 and 2.

**Table 1:** Demographic description of Focus group participants in Group 1.


PARTICIPANT NUMBER	CLINICAL SETTING	PROFESSIONAL QUALIFICATIONS	YEARS QUALIFIED	REGION	REFRACTING EXPERIENCE

One	hospital	orthoptist	10–30	London	over retinoscopy

Two	hospital	student orthoptist	N/A	Scotland placements UK wide	university based teaching and some on placement

Three	hospital	student orthoptist	N/A	North-East England placements UK wide	nil

Four	hospital and private	orthoptist and prescribing optometrist	10–30	South-West	some as orthoptist core as optometrist

Five	hospital	student orthoptist	N/A	Scotland placements UK wide	university based teaching and some on placement

Six	hospital	orthoptist	<10	London	nil

Seven	hospital & university	optometrist	10–30	London	refraction on every patient


**Table 2:** Demographic description of Focus group participants in Group 2.


PARTICIPANT NUMBER	CLINICAL SETTING	PROFESSIONAL QUALIFICATIONS	YEARS QUALIFIED	REGION	REFRACTING EXPERIENCE

One	hospital	orthoptist	10–30	London	nil

Two	hospital and community	orthoptist	10–30	Scotland	nil

Three	hospital	orthoptist	>30 years	South-East	retinoscopy (dilated, undilated, over refraction, Mohindra, Bruckner, dynamic)

Four	hospital	orthoptist	10–30	South-East	paediatric cycloplegic refractions

Five	university and community	prescribing optometrist	10–30	North-West	all types, core technique

Six	community practice	orthoptist prescribing optometrist	10–30	London	core technique in practice

Seven	community	orthoptist pre-registration optometrist	10–30	London	as orthoptist: in research as optometrist: core technique

Eight	hospital	orthoptist	10–30	London	nil

Nine	hospital	student orthoptist	N/A	North-West England	learnt as part of degree


The focus groups were conducted online via Microsoft Teams Version 1.3 (Microsoft Corporation, 2017). Remote video conferencing was chosen to facilitate data collection and to allow clinicians from across the UK an opportunity to attend (Kite and Phongsavan, 2017). Both audio and video recordings were used to capture verbal responses and observe body language (Tuttas, 2015). The researcher (MC) remained present but did not contribute to the discussion. The facilitator (RS) ensured a comfortable environment, outlined the research question and encouraged all participants to contribute to the discussion. The facilitator employed both semi-structured questions where participants describe their experiences relevant to the research question and open-ended enquiries to gather more in-depth information. Focus groups were limited to a one-hour time constraint which was based on estimates of around 6 to 10 participants (Tuttas, 2015). Research suggests that this length of time increases participation and reduces fatigue (Gray *et al*., 2020). Recordings were made with Microsoft Teams software. All participants were informed when the recording began. Transcripts were generated using Microsoft Speech Services, then reviewed for accuracy by MC. Comments from participants were anonymised. Transcripts were analysed using NVivo 12, employing thematic analysis to identify patterns.

During data analysis, two researchers (MC and RS) took notes on codes and participant agreements to aid interpretation. Thematic analysis adopted the following steps:

1. Transcripts were reviewed by the principal researcher for accuracy.
2. Both researchers involved in coding, familiarised themselves with the data. Each transcript was independently coded by two researchers, analysing each sentence. They created a list of codes including their definitions to ensure consistency in coding. Refinement and consensus were reached through discussion.
3. Codes were collated into developing themes. Themes were divided into sub themes wherever appropriate.
4. All data were checked to ensure consistent and coherent grouping within each theme. Participant identities were anonymised in all the quotes.

## Results

The main themes and subthemes that evolved from both focus groups are presented in Table 3.

**Table 3:** Main themes and subthemes from focus groups.


MAIN THEMES	SUBTHEMES

**To what extent are orthoptists currently refracting?**	Responses indicating some orthoptists refract.

	Responses indicating that refractions are mainly carried out by optometrist or ophthalmologists.

	Responses indicating that it can be difficult to find an optometrist or ophthalmologist to work in refraction clinics.

**If participants have experience of orthoptists refracting, how do they prescribe and how are they supervised?**	Responses from orthoptists who undertake retinoscopy with consultant signing prescription.

	Response about multi-disciplinary team involved in optical prescribing in the HES.

**When orthoptists undertake refractions, in what setting does this occur?**	None.

**Training for orthoptists in undertaking refraction**.	General responses about training.

	Responses about changes in orthoptist training in refraction over the years.

	Responses about opportunity to practise refraction.

	Responses about the need for training and appropriate pathways.

	Comments that the training orthoptists receive in refraction is comparable to that of ophthalmologists.

	Comments about variability in training between clinicians.

**When orthoptists undertake refractions, what are the results used for?**	Aid clinical decision-making.

	To prescribe (optical prescriptions).

	The importance of future prescribing practices for orthoptists.

**Potential disadvantages and mitigations to orthoptists undertaking refractions**.	Patient safeguarding.

	The need for patients to be under a consultant ophthalmologist.

	The need for orthoptists to have sufficient training.

	Need for a regular ocular health assessment.

	Concerns about commercialisation if orthoptist were able to issue optical prescriptions.

**Potential advantages to orthoptists undertaking refractions**.	Aid clinical decision-making.

	Reduce commercial pressure and improve clinical care.

	Cultural changes in eye care.

	Dynamic retinoscopy.

	More cost-effective.

	Patients would prefer one appointment.


### 1. To what extent are orthoptists currently refracting?

Seven respondents indicated that they were either an orthoptist who was already performing refractions and/or that they knew of orthoptists performing refraction (Table 4). Other responses indicated that refraction was mainly carried out by other clinicians such as optometrists or ophthalmologists and that it was more unusual to have orthoptists carrying this out. Some responses also highlighted challenges in finding clinicians willing to carry out refractions.

**Table 4:** Examples of responses to question: To what extent are orthoptists currently refracting?


THEME	EXAMPLE RESPONSE

**Responses indicating some orthoptists refract**.	I know of orthoptists who refract on behalf of consultant ophthalmologists.

	xxx was this trailblazing orthoptist that refracted.

	There are a couple of orthoptists. They do. They do refraction clinics.

	So, I refract. I’ve been refracting for quite a while now, and another orthoptic colleague refracts.

	Myself and my colleague, had an interest in refraction we have attended one of xxxxxx courses, in the past and we’ve been trained in-house ‘it’s all under the consultants.

	There’s a lot of orthoptists already refracting.

	I know there are departments around England where orthoptists are doing retinoscopy.

**Responses indicating that refractions are mainly carried out by optometrist or ophthalmologists**.	I think most of our ophthalmologists refract in paediatric ophthalmology clinics. Where in previously I worked in GDHs (General District Hospitals) it was more optometrists that would do the refractions in those clinics.

	I’ve never seen an orthoptist do refraction, they’ve always been sent through to an ophthalmologist or an optometrist.

	I’ve only seen optoms doing it or occasionally the ophthalmologists.

	There are no orthoptic refractions happening in Scotland and I think I think, because it’s still not the norm, I think.

	The referral usually comes from the orthoptist, wanting me to see the patient. So, I don’t think they actually know they do refraction themselves.

	But I don’t think it’s a thing the orthoptists are generally doing.

	But I think that goes to show you just how limited certainly, from my experiences of working with orthoptists that refraction is done by orthoptists.

	Again, it’s not the norm to have orthoptists refract.

**Responses indicating that it can be difficult to find an optometrist or ophthalmologist to work in refraction clinics**.	We do not have a lot of optoms on our team.

	When we’ve got big backlogs and that’s how it came about.

	And you can’t get hospital optometrists.

	But we have trouble recruiting optometrists to refract children.


Note: When participants mention “optoms,” they are referring to optometrists.

### 2. If they had experience of orthoptists refracting, how do they prescribe and how are they supervised?

Three responses indicated that orthoptists perform retinoscopy with the agreement of a patient’s consultant who would then sign their prescription. Four stated that the final decision about optical prescribing is already a multi-disciplinary decision and that team members need to discuss and decide what is best for their patients (Table 5).

**Table 5:** Responses to question about how orthoptists prescribe (optically) and how they are supervised.


THEME	EXAMPLE OF RESPONSES

**Responses from orthoptists who undertake retinoscopy with consultant signing prescription**.	I know of orthoptists who refract on behalf of a consultant ophthalmologist and then the ophthalmologist will prescribe the glasses based on the findings of the orthoptic refraction.

	I do retinoscopy with the, you know, the agreement with the consultants. And so, you know, everything is all you know, signed off and agreed. But I’m the one who does the retinoscopy.

	I know that in some departments they work with the agreement and the signatures of the consultants for the prescription of glasses.

**Response about multi-disciplinary team involved in optical prescribing in the HES**.	And then from that because it’s a small department, we’ll talk to each other.

	And so, we sort of discuss that amongst ourselves.

	That largely depends on what clinic, or if I’m working with somebody else, including, of course, that would be a joint decision.

	Yeah, it’s joint decision-making.


### 3. When orthoptists undertake refractions, in what setting does this occur?

Comments indicated that orthoptic refractions are presently performed in a National Health Service (NHS) hospital environment. The participants also felt that there was no desire from orthoptists to work in community optical practices and that orthoptic refractions should always be limited to the hospital environment (Table 6).

**Table 6:** Responses about the setting in which orthoptists refract.


THEME	EXAMPLE OF RESPONSE

**Responses about the setting in which orthoptists refract**.	I just wanted to clarify exactly which bit we were talking about and in a hospital environment.

	But having that orthoptic background and maybe doing some paeds (paediatric) refractions, especially when we’ve got such a backlog in the NHS.

	They want to come into the hospital and do glaucoma clinics, retinal clinics work in the acute high service to get independent prescribing.

	I mean I wouldn’t know of any other setting than a hospital. My experience is that orthoptists primarily work in NHS Trusts.

	It is a hospital setting yes.

	I don’t think as a profession we would be keen to be working in a high street practice.

	I don’t think anybody wants to go out into the high street and start refracting the children.

	Orthoptists are extremely well placed within the hospital setting for cycloplegic refractions.

	I know very few orthoptists work independently on the high street.

	I don’t want orthoptists going out into the high street, saying. You know I can prescribe anybody glasses, and I’m going to set up my own shop, you know, and I don’t think we would particularly want that. There is definitely a role in hospital practice.

	There is a clear indication for all of orthoptists to refract within the hospital eye service.

	And I think that’s the advantage of having the hospital setting under consultant care.

	Can I just say that going back to being restricting in the hospital setting and the community setting, I think, would be ideal. [participant was supporting the fact that orthoptists should only be allowed to refract within a hospital environment].


### 4. Training for orthoptists in undertaking refraction

Having listened to the discussion, the clinicians’ who had been qualified the longest acknowledged that it was likely that orthoptic students now receive more training on refraction, compared to when they were a student (Table 7). There was significant discussion indicating that refraction is not a skill that a newly qualified orthoptist would be confident in applying. Rather, they would need a significant amount of practical experience in this area to be allowed to perform refraction as part of an extended role. There were comments that orthoptists receive comparable training in refraction to ophthalmologists, but only ophthalmologists (or optometrists) are legally allowed to issue optical prescriptions. There were also observations that if the law was to change to permit orthoptists to issue optical prescriptions, then orthoptic training should be better than what ophthalmologists currently receive.

**Table 7:** Responses about training for orthoptists in undertaking refraction.


THEME	EXAMPLE RESPONSES

**General responses about training**	We have been trained at uni so like ret and stuff. But on placement. We’ll follow the child through, and then they’ll give us a go at doing it ourselves.

	Optics in year 1 optics and year 2 and more practical refraction year, 3. So probably 3 modules based on optics or refraction. And the cumulative of that would be, we had to do a refraction on 2 adult patients in clinic. There was dry ret and a subjective refraction afterwards and that was probably what I learnt as a student.

	I also did optics in year 1, year 2, and a refraction in year 2 as well.

	We are taught to refract at university, which is great. But refracting in a university clinic room with your peers isn’t the same in a hospital, and if someone told me. Once I had graduated to go and start refracting patients. I think I would scream because I wouldn’t know what I was doing.

	The course that I run, the course was entitled Non-Prescribing Retinoscopy Skills. It was to talk about non prescribing skills. It was about the mechanics of retinoscopy. It was particularly about dynamic retinoscopy and the Mohindra retinoscopy, and over refraction.

	We have actually attended one of xxxxx courses in the past and we’ve been trained in-house it’s all under the consultants.

**Responses about changes in orthoptist training in refraction over the years**.	Yeah, just about remember my orthoptics degree it was about 25 years ago. Now. So, bear that in mind. But yeah, we did. In our final year. We did have a brief module in refraction it was. We had an optom come over from those hospital in Manchester. I think I just did some afternoon sessions. So, it was piecemeal. It was like a taster like we had a taste in how to use a slit lamp and a taste in various other kind of the sort of things that you might encounter in in eye testing.

	So, maybe orthoptists are getting more retinoscopy practice. I hardly had any when I was an orthoptist student. And then thinking back to my university when doing optometry, there were lots of labs where we would practise refraction. So, I think after the degree, you do have a lot more retinoscopy experience as an optometrist.

**Responses about opportunity to practise refraction**	But mainly it’s just some exposure in joint clinics when the patients are going through, like XXXXX said and they do know that we’ve been trained as well. So, occasionally we get the opportunity to participate.

	My main thing is that the skills involved in refraction aren’t just about prescribing glasses, and I think that orthoptists are taught retinoscopy and subjective refractions and the optics, and they get it in uni and they come out probably at a level equivalent to a newly qualified optometrist, but they just don’t, often they don’t get to practise it so often. Those skills are lost.

	So we have an optics module in year 1 and year 2, kind of the theoretical side, and we did refraction in year 2 in clinic, and we ended up doing, I think, I don’t know if it’s because of COVID, but we didn’t end up seeing patients to do refraction on. We did have an exam, though that incorporated having to do Ret in year 2.

	COVID sort of had an impact. So, it was like model eyes, and they were very creative. But unfortunately, it wasn’t patients from out in the street. So, we were just doing it on each other.

	I think we do get patients from like the BV Clinics and things like that as well as doing on each other. But I think not in first year. It’s still model eyes in first year. But I think in second year you are supposed to get patients it’s just we never got that opportunity.

**Responses about the need for training and appropriate pathways**.	And I’ve heard of a refraction, and like possibly extended roles or something like that.

	I think it’s all about how much training an optom gets or an orthoptists gets, or you know that how much time is devoted to that to provide the training because it there is a lot of clinical experience that’s needed.

	I don’t want to mention it, but you know you’re not very good at things you don’t do very often. So, if you’re going to go down that road, you kind of need to make sure it was part of your workflow, I think just doing it very, very occasionally is not going to be great. My only caveat in the back of my mind is in my degree. I think you get a taster of what refraction really is and I don’t think many orthoptists who qualify, would feel super confident to do refractions straight out of the gates.

	Worked it out about 650 a year, or something like that, so, I do quite a few of them and there are certain types of lens which have these really weird split reflexes, and you have to do a lot of them to be able to work out which bit of that reflex you’re looking at. So, an auto-refractor is going to get caught up with that. Someone who does ret once the month is going to get caught out on that. A lot of optometrists, who, in fact, refract a lot of the time get caught out with that sort of thing because they’re not doing a lot of them. So, it’s that getting good at it here you got to do the numbers to get good.

	Yeah, but I again, I suppose it’s not to say that people shouldn’t do a test because they’re not going to get good at doing it, because then you’ll never get good at doing it.

	I don’t think we are all competent to refract. Of course, there is going to be extra training, practice, and things come into place.

	There are analogies where people work. They have a degree of qualification in prescribing drugs but there’s still someone else that owns that decision, and they have been signed off as competent to make those sub decisions, if you like, as part of the care package.

	I work there we weren’t allowed to independently prescribe ourselves. The ophthalmologist had to sign off every prescription. That was just the rule in the hospital. So, you would write a prescription out for any patient, and they would look at it, and often I don’t know they would change it, depending on what they thought. You know they would ask for a re-refraction. But you know they then decided that it was up to us as optometrists to do what we wanted, and then we were given those rights to make decisions ourselves and it is difficult. I remember the first few months; you know you didn’t have that person, oh, sitting on top of you signing everything off, so, you got independent pretty quickly, but it was because, you know, in the refraction clinic we do 40, 50 refractions a day. It was extremely busy and you just learnt on the job doing things really quickly. So, I think you definitely, you know, if you are thinking of being good at refraction, you do need that experience.

	Which is where the pre-reg (pre-registration) year in optometry is really useful, because you get that experience but orthoptists don’t have that pre-reg (pre-registration) year, and if you’re going to learn refraction fairly late on in your course there is not going to be the number of patients to see, to sign off to say, yeah, okay, you know you understand why you don’t give this in that situation. Why, it’s beneficial to get that in that situation. So, it’s again. It’s the just the numbers.

	So, I think, without the volume of knowing what is normal and the training, of the disease without all of those tendencies being in place. It. It’s just going to take one thing to go wrong, and it just it becomes futile really. So, that would be it.

**Comments that the training orthoptists receive in refraction is comparable to that of ophthalmologists**.	But compare our training to what ophthalmologists get when they do their part one training. I think it’s probably comparable, so I don’t think ophthalmologists have an awful lot more training. But they are allowed to prescribe glasses and I find that it will be a junior ophthalmologist that’s more likely to come to me and say, what do you think we should give here.

	When the Royal College of Ophthalmologists have their DO (diploma in ophthalmology) exam, and they have a refraction exam which is quite basic. You don’t have to be terribly good to pass it. To be honest, I mean I think we would aim for much higher standards than the Royal College of Ophthalmologists

	I taught on some of those courses, and well, let’s just say that one of them wants to prescribe ten base down on an asymptomatic patient, because the trial frame is a bit wonky. Look we get ophthalmic medical practitioners that might be GPs refracting on the high street and they you know again, there’s a few good ones, but it’s not generally the best use of them.

	We would aim for much better than that.

**Comments about variability in training between clinicians**.	I think that orthoptists are taught retinoscopy and subjective refractions and the optics, and they get it in uni and they come out probably at a level equivalent to a newly qualified optometrist, but they just don’t. Often, they don’t get to practise it so often. Those skills are lost.

	That orthoptists come out with the same level. I kind of disagree with that, because in optometry. I think it’s a long time ago, and so maybe things have changed a bit.

	So, maybe orthoptists are getting more retinoscopy practice. I hardly had any when I was an orthoptist student and then thinking back to my university when doing optometry. There were lots of labs where we would practise refraction. So, I think after the degree, you do have a lot more retinoscopy experience as an optometrist.


Note: When participants mention “optoms,” they are referring to optometrists; “uni” refers to university; “ret” refers to retinoscopy; “GP” refers to General Practitioner; and “BV” refers to binocular vision.

### 5. When orthoptists undertake refractions, what are the results used for?

Four responses indicated that orthoptists use the results of their refractions to aid clinical decision-making to improve the management of their patients and make appointments more efficient in terms of time and costings within the hospital (Table 8). Two responses indicate the results of the refraction are being used to prescribe spectacles. One response indicated that they were delaying annual refractions in their department, as there were no available appointments. This person also stated that certain types of patients don’t need another refraction instead they just need some extra plus in the form of a bifocal.

**Table 8:** Responses about uses of the results of refractions undertaken by orthoptists.


THEME	EXAMPLE RESPONSES

**Aid clinical decision-making**.	Orthoptists may do retinoscopy, or may refract, but not for the purposes of prescribing glasses. But to inform your practice. So, if you’ve got a patient, you can maybe do, and you’re not too sure if they need to have a refraction again or not. You might do a bit of over-ret (over-retinoscopy) just to make sure you’re fairly happy with what the glasses are at. An imbalance with the vision, and that might inform kind of when you’ll book them back to see the optom.

	But essentially for myself personally, I will pick up a ret. But I’ve not got the intended idea of doing a refraction with a view to prescribe glasses. It’s a ret just for your information, you know. If you’re screening a child you might just ret and say, do you know what, you might just think you are myopic or probably myopic because they are usually dry just to give you a bit of background.

	I think if well, if there’s a result, it will definitely help on any clinical decision-making.

	It was just about what the refraction results are used for, whether they use for prescribing or kind of your clinical decision-making from an orthoptic perspective? I mean both. Really, I think, I think I keep talking about this with paeds in my mind. But actually, there’s like there are other clinics where I will want the results of the refraction, but I won’t necessarily be looking at prescribing. So, if it’s an adult strab [strabismus] clinic, for example we tend not to give adults glasses in the hospital service. Primarily because of the cost. So, the patients should be collecting them from their own optician. But I want the results of that refraction test. Because I’m not worried about amblyopia necessarily, but I am worried about your BV (binocular vision). So, if you’re minus 2 and plus 2, and you can see alright, But actually, you’ve got a decompensating phoria. Then I want to know actually if it there’s a refractive, cause, behind that. So, I’m not always interested in prescribing. But I’m often interested in what the refractive error is, and I think adults strab is a good example of that, and also a lot of orthoptists now like we do our department. We have an orthoptic led low vision service here. So, the orthoptists do the low vision clinic again the optoms say go get your glasses from the optician shop or from your local optometrist. We did the low LVA [low vision aid] dispensing here. So, we’re not necessarily going to prescribe from that. But I want to know that your glasses are roughly right in order to do that.

**To prescribe (optical prescriptions)**.	We have optometrists that they tend to do the new cases, and I will do follow ups. But we all agree.

	That largely depends on what clinic, or if I’m working with somebody else, including, of course, that would be a joint decision. But if it’s only an orthoptic clinic maybe another, orthoptist, I’m not. I’m not really confident enough, or don’t really know what I’m doing the right thing, or if I have, if I think I have the knowledge and the confidence. I wouldn’t give a straight away answer.

**Responses about the importance of future prescribing practices for orthoptists**.	We are just so short on the refraction clinics at the moment. To the point that, actually, we’ve had to consider delaying annual refractions. If the vision is okay and obviously document exactly why we’re doing that just because we don’t have the capacity and saving those slots for those that really really need it. And yeah so, I think on the other aspect. Actually, if you know, we’ve got those children, maybe a convergence excess that, you know, needs a bit of plus on their bifocals. You’ve got the accommodative squints there that they do need looking at really from an orthoptic perspective. So, it would just be really handy for an orthoptist, just to be able to step in and have a look at that.

	From the adult’s point of view is, I think there are a small, there is a small, cohort of patients that actually do probably require hospital refractions. In terms of you know the stroke patients that we see. So, a lot of the time we are saying to them, you know, once you’re discharged that your vision’s a bit down. We’ll see them at the bedside their vision is a bit down. Go home. Once you’re home book a domiciliary visit? But a lot of the time. I think there is probably a need for some of those patients to actually be refracted in the hospital. Whether it’s subjective, or you know, whether we even cyclo (cycloplegic refraction) then if they can’t manage it.

	It’s just general kind of amblyopia management it’s just it would change things, I think, when you need to check somebody’s refraction before that annual one-year time is up before you go and change your amblyopia treatment. Or you want to see if the refractions changed, or something else is going on. It would just be such a quick tool rather than booking them in to see an optometrist, you know you just got a lag or a delay. I think, from an orthoptic perspective, you’d be able to tell whether this is a big enough change that warrants you know a prescription change, or can you just go ahead with changing, say, an occlusion regimen, things like that? And I think that would save a lot of the appointments as well, just waiting for an optom if an orthoptist could just check and then just deal with their management as well.


Note: When participants mention “optom,” they are referring to an optometrist; “ret” refers to carrying out retinoscopy; “paeds” refers to paediatrics; and “strab” refers to strabismus.

### 6. Potential disadvantages and mitigations to orthoptists undertaking refractions

There was a discussion that if orthoptists are to play a greater role in refractions and issue optical prescriptions, this would need to be carried out very carefully to safeguard patients. Participants either quoted examples of where orthoptic led refraction clinics were already running successfully within a hospital environment or where clinicians felt refraction clinics would be allowed if they met the following requirements: the patient was under a consultant, in an NHS environment, and with satisfactory health checks in place. There was general acknowledgement that there would need to be sufficient training to ensure patient safety. Responses from the focus groups stated that it was very important for patients to have a regular ocular health check, in addition to the refraction, particularly in a hospital environment where there is more likely to be pathology than in the community setting. The final worry that clinicians had in relation to orthoptists playing a wider role in refractions is that orthoptists should not be allowed to carry out refraction in community optical practices (‘on the high street’) (Table 9).

**Table 9:** Responses concerning potential disadvantages to orthoptists undertaking refractions and mitigations.


THEME	EXAMPLE RESPONSES

**Patient safeguarding**.	To be able to do dynamic retinoscopy to formulate your clinical decisions as an orthoptist I think that’s an excellent idea and I would very much encourage that. To let all orthoptists, prescribe spectacles on the basis of refractions of their own refraction results, I think that is extremely dangerous.

	I came in here thinking it wasn’t a good idea, but I am thinking it is, but I think there has to be safeguards in place.

	And as things stand at the moment, you’re giving that to say, I sign off this person’s eye health and these numbers, and I think that there’s got to be. Where’s that ownership come from?

**The need for patients to be under a consultant ophthalmologist**.	And I think they it’s very dangerous potentially to be looking at refraction as just a thing where you put some lenses up, and you give some numbers, and you give it on a bit of paper for some specs, because it’s not. It’s all part of that sight testing the whole routine, if you like the whole kind of comprehensive examination of the eyes. That’s where clinics, for example, XXXXXX where she was doing the refractions they still had to be. Every patient had to be signed off by a consultant.

	But I think when I’ve been thinking about it, I’d be happy if it was allowed under certain circumstances, in a hospital setting like xxx says that you know you have access to the notes, so you can look at the fundus findings, or you know, if you feel there’s a problem at the back of the eye. You can take them to an ophthalmologist or an optometrist and ask them to take a look so. You are in that kind of supportive system.

	Would have been signed off by a consultant who would have allowed her to do the prescribing like to write the prescription because she did write and sign it.

	So, prescribing to issue an optical prescription. Yes, but I think it has to be a patient that is, under the care of a consultant ophthalmologist like most children are in in orthoptic departments. There has to be some safeguard that someone looks after the eye health and has responsibility for that.

	So, all the paediatric patients will have an Optos, and that will be reviewed virtually by whether it be an orthoptist and optom, a doctor who is qualified to do that. So, you know all the children get a full eye health, check and the orthoptist would not see currently a new patient.

**The need for orthoptists to have sufficient training – students**.	I think that on placement there is just not an opportunity to even really go and watch either, that I’ve come across yet.

	I mean, we’re learning about it in uni and you need so it’ll be great to use it like whilst we work. I do agree with what xxxx said, though a wee bit ago, and that you would have like to get really good at something. You have to do it really, frequently.

	I think in an order for us like to be good at it, and be confident, and it would need to be a sort of regular thing.

**The need for orthoptists to have sufficient training – qualified orthoptists**.	And there’s a this, the art, not the science behind it, and that’s the only thing you have to do a lot of those things to get good at doing.

	I think it would. It would be an extended role like a lot of orthoptics extended roles. I don’t think it would be a newly qualified core skill.

	Somebody told me that you had to do a 1,000 retinoscopy episodes before you felt confident, and once I had done that, I knew what they were talking about, so it’s not a newly qualified skill. It’s something that you need to get lots of practice. And then you need quite a lot of with cycloplegic retinoscopy particularly. Whoever’s making that prescribing decision hasn’t got the security blanket of a subjective response. They basically just have to go for it. And so, I think hospital optometrists and consultants are used to saying, this is the best I can do. We’re going to go for it, and I think it does take a lot of clinical confidence. And so, you need to have done lots, so I don’t think it’s a newly qualified skill.

	Think it’s definitely an extended role, and I think there are plenty of ways of quality assuring it, and it’s not just getting 50 within 50 you know of 0.5 diopters. It’s a matter of managing a challenging case. You’re only aiming to be within a diopter on a non-challenging, case you’ve got to be spot on, and there are sort of ways that it could be done and I think it’s quite possible to do it, and I don’t think as a profession. We specifically want it to be a newly qualified skill, but I think it could well be an extended role.

**The need for regular ocular health assessments**.	I was gonna add I honestly, I’ve got no problem with orthoptists refracting. Obviously, we have to have that some kind of health check there, because there’s a lot of eye disease in in the hospital

	That’s my big worry is about the again is that the eye health assessments which are integral to that number on that sheet of paper

	I sign off this person’s eye health, and these numbers, and I think that there’s got to be. Where’s that ownership come from?

	I believe a refraction is done to find a problem and not just to give glasses. So, I think if an orthoptists is. I’m not saying that an orthoptist is not looking for pathology, but there are certain pathologies that you know, an orthoptist would not be trained to look at.

	We (orthoptists) are already doing AMD injections. We’re already doing all glaucoma management glaucoma screening all sorts of other things. So, we used to. Now they are getting very highly trained. They might not have up to date experience. But the courses are getting improved and putting a lot more ophthalmology in. So, I think it’s definitely a follow-on qualification. But actually, they are trained to examine and look at pathology in quite a lot more detail than they used to be. You know that students might not feel terribly confident in it. But then this isn’t a newly qualified skill. So, I think there are ways that it is already being managed for fundus checks and that sort of thing.

	So, all the paediatric patients will have an Optos, and that will be reviewed virtually by whether it be an orthoptist and optom, a doctor who is qualified to do that. So, you know all the children get a full eye, health, check and the orthoptist would not currently see a new patient.

	Just a question, because I am not 100% on this. But I think in Australia orthoptists refract. But they don’t do fundus checks. That’s how I understand it and I wonder what the patient sector data or equivalent is which says whether or not this works.

	But it’s not just Australia.

**Concerns about commercialisation if orthoptists could issue optical prescriptions**.	Can I just say that going back to being restricting in the hospital setting and the community setting, I think, would be ideal. I would be concerned that there is crossover, for orthoptists entering, high street practices and refracting because that could also be utilised by multiples to see the younger children but not necessarily with the same level of care.

	I think one of the biggest reasons hospitals struggle to recruit optometrists is they just don’t pay the amount that you can get in the community. Even many community optometrists are stopping doing NHS eyes tests, because the NHS will pay you £22 roughly for an eye test. So, they will all need to see private patients now, and that’s happening probably more and more now. And so, the concern would be if orthoptists were given the ability to prescribe issue prescriptions for glasses. Without the appropriate sort of safety checks in place you will see some orthoptists leaving moving to the community where they would earn a lot more, the multiples would yeah take advantage of that. And orthoptists there would be some that would leave and leaving the NHS in a bigger dilemma.

	We don’t want to train orthoptists to then go off into the High Street, and just prescribe myopic prescriptions or issue you know myopic prescriptions any more than you want us doing that. And I think it’s just a matter of getting together and drafting something legal that satisfies both professions.

	No, I’ve already said. The thing that I think is a disadvantage that I am worried that it will be used inappropriately. Abused by people to make money, and that is not well. It’s not not what I want to see happen.


Note: When participants mention “Optos,” they are referring to a retinal imaging device; “uni” refers to university and “optom” refers to an optometrist.

### 7. Potential advantages to orthoptists undertaking refractions

There were six responses about how orthoptists carrying out refraction could improve clinical care (Table 10). There were some specific examples of binocular vision cases where knowledge of the patient’s current refractive error is required to manipulate their prescription and manage the patient appropriately. In addition, there were several generic comments that knowledge of the refractive error is essential to ensure that the management of the patient’s binocular vision anomaly is based on all relevant information, which includes an understanding of their refractive error. Some clinicians felt that by carrying out refractions in a hospital environment there could be less commercial pressure which would improve clinical care. One comment acknowledged that patients with special educational needs or autism may struggle to find an optometrist who is willing to see them or to receive the correct treatment. There were two comments relating to the fact that ophthalmologists no longer want to or should be asked to refract. Multiple comments stated there are not enough optometrists willing to work in hospital paediatric refraction clinics. There was a feeling that some optometrists preferred clinics that dealt with pathology such as glaucoma. Another popular theme running through the focus groups was that some optometrists and ophthalmologists might be becoming deskilled in carrying out retinoscopy, suggesting that auto-refractors and pre-screening (by optical assistants) might be partly to blame for this skill becoming redundant. Four comments indicated that some optical practices refuse to see younger children. There were several comments that many clinicians don’t know how to carry out dynamic retinoscopy. There was a comment that because of the way the hospital clinic is set up clinicians are unable to carry out the technique. There was also a comment that patient care would improve if they could measure accommodation. There were comments that if orthoptists could issue optical prescriptions, this would be cost-effective to the NHS by allowing orthoptists to carry out refractions instead of ophthalmologists when optometrists are not available. The final potential advantage cited was that patients might prefer to have fewer visits to the clinic.

**Table 10:** Responses concerning potential advantages to orthoptists undertaking refractions.


THEME	EXAMPLE RESPONSES

**Aid clinical decision-making**.	In your day-to-day orthoptic life you have got a patient who’s in bifocals for example, for your convergence excess and you are trying to train them out of this bifocal. What’s really useful you know, is then, is being able to say, you know your refraction. You have only been refracted six weeks ago. The refraction’s not changed, but we want to start bringing you out of this bifocal and the practicality of doing that, in an orthoptic clinic with no optometrist is really difficult. So, if we could you know, using clinical judgement and professional etiquette you would take that base refraction and you would at least manipulate your part of the bifocals in order to manage the squint.

	Or, you know, you have got somebody you know their refractive error you have got them on some minus lens therapy. Again, you don’t want to try and tear them out their minus lenses therapy well we can’t do that without. So, you come to the orthoptist to manage your squint, but then we end up booking back, depending on your setup to the optometrist to reissue the prescription, and actually that is a scenario where you want the prescription. But you don’t necessarily want to repeat refraction because it’s recent. It’s valid. You’re happy with it. We’re just doing that bit where we manipulate lenses to help control the squint.

	As an orthoptist I definitely think that if you have the knowledge, because the refraction is not only numbers, because the knowledge will tie in into all the diagnosis and all the symptoms, and it will definitely deliver a more well-rounded patient care.

	It hasn’t made sense to me why orthoptists aren’t allowed to do a refraction in certain circumstances, because you know, when you are doing a BV evaluation, you do need to understand what the proper prescription is, and I’ve never quite understood how you can do proper care if you don’t have the updated prescription. So, I would always like to make sure the patient has the right prescription, or you know, if you are trying to modify the prescription in some way.

	So, I think it’s really necessary to have that up-to-date prescription, and I’ve never worked out, why, you know, orthoptists haven’t been allowed.

	I’m sure there are many advantages, and, as you say, the patient experience. And again, looking at the non-prescribing bit of refraction, I totally think that’s a great idea, because then the patient gets that holistic view.

**Reduce commercial pressure and improve clinical care**.	But there are lots of optometrists that are only interested in throwing as many people through in the day as they can, and nobody really cares about binocular vision. I can talk to them until I’m blue in the teeth about the fact that so you can find that somebody’s a +3. But as the kids that I had today who’d been prescribed +3 was then exotropic with it unless you’re going to do the cover test etc., afterwards. Well, actually, it’s meaningless because that those bits are an art. So, this poor kid was eso (esotropic) without his glasses o’ his prescription is +3, but because he’s a bit older now he doesn’t need all of that, and that was the result of his double vision. But he’s been round lots of different places and in all honesty an orthoptist would have spotted that straight away.

	Interestingly, we’ve had a fairly newly qualified optometrist recently join our team. He’s been in retail for like 2 years, but he felt like XXXX was describing like everyone is almost pre-checked, and there was this pressure of how many can you get through the door and there wasn’t really much time, especially with the younger children, to take out that ret and see them properly, as you would want to.

	So, I think it’s coming back to this. If you are in a busy high street clinic, and your fee model depends on the number of pairs of spectacles. You see, you don’t want to see anybody who’s anything other than either a –2 where you want to change it. Or oh, look, you need another 0.50 [half dioptre] on your varifocals because there’s no incentive. Now, not all optometry practices like that. So, lots of people aren’t for example. But I honestly, I would have no problem with orthoptists refracting at all.

	I have more of an issue with this, and I know this wasn’t the question, but with DOs refracting and the reason for that is. It is going to be hijacked by the multiples to get more and more people through and then we’ve got this.

**Cultural changes – Patients with special educational needs or autism struggle to find an interested optometrist**.	So, lots of people will just go. We’re not going to see them, because (a) we might not sell them glasses and (b) I might have to cyclo (cycloplegic refraction) them, but there are certain areas now, and it’s a patchwork of there will be a pathway in place. So, if the child comes with x, y or z there’s a certain fee for doing the cycloplegic refraction. But I think the issue is that, as everybody said. Seeing kids and seeing people with special needs is a skill, and you need a volume of it I don’t think well, certainly in XXXX there’s not lots and lots of people desperate to see these kids, which is why, again, I’m getting them coming down from XXXXX. That’s ridiculous. You shouldn’t have to drive your kid 50 miles to see an optometrist who’s actually going to be a bit interested in them. So, there are pathways, but it’s patchy.

**Cultural changes – Ophthalmologists don’t want to refract**.	Paeds consultants now don’t like to do as much of their refractions as they perhaps they used to be doing.

	And it’s just really not appropriate to be booking these all into the doctors’ clinics.

**Cultural changes – Difficulty finding optometrists to refract in paediatric hospital clinics**.	Because I think previously. We do not have a lot of optoms on our team and people just work in different days.

	Whereas a lot of optometrists are actually keen to get on with doing other stuff that hospital optometrists particularly they are very keen to get on with other extended roles.

	You know sometimes there are lots of optometrists who they do it, and in other places they aren’t. And you can’t get hospital optometrists.

	It came about because we basically didn’t have enough refraction clinics, we have at the time we had eight or optometrists that work within the unit. But we have trouble recruiting optometrists to refract children. They want to come into the hospital and do glaucoma clinics, retinal clinics work in the acute high service to get independent prescribing.

	So, you know, we’ve just got a backlog. We can’t get the optoms, so the way forwards for us is to train up the orthoptists.

	Certainly, in my own Health Board we have two paediatric consultants and we have in-house optometrists, and we have really struggled to recruit in house optometrists once somebody had retired.

	The reality is that optoms coming out are not really interested in these special roles. They want to do more medical glaucoma other stuff like that.

**Cultural changes – Orthoptists’ skills are better than most optometrists for prescribing in children with binocular vision anomalies**.	And BV and cyclo has always scared, I think, optoms. That’s from my personal experience. So, I think orthoptists refracting is a good idea.

**Cultural changes – Clinicians becoming deskilled at retinoscopy**.	I have had word from the XXX for anonymity that retinoscopy skills are a dying skill in optometry.

	I just say that I completely agree about the optometrists, not being able to do retinoscopy anymore.

	So, it annoys me that optometrist don’t do it because I just think, well, why? Because you learn so much, but it’s because it’s because all the pre-screenings done for them. There they put everyone. Sorry I know it sounds like I’m generalising. But most people go on an auto-refractor. So, you’ve You have got kids, so they they’ll will cyclo (give them cycloplegic drops) them and stick them on an auto-refractor.

	It’s the only one day a month but you still need those skills there, so I agree it’s a dying art.

	He’s been in retail for like two years, but he felt like xxxxxx was describing like everyone is almost pre-checked, and there was this pressure of how many can you get through the door and there wasn’t really much time, especially with the younger children, to take out that ret and see them properly, as you would want to, and, interestingly, he was probably the only one who interviewed to say that actually I really enjoy taking the ret out and actually looking myself, which is really good.

	I have lots of pre-registration optometry students come through to my practice, and with lots of optoms that don’t routinely do cycloplegic refractions or any paediatric refractions and you can see the impact of what they’re going to give what it would be, how they make that final decision.

	Lots of times the consultants will ask for an autorefraction. And that’s done by the nurse, or sometimes the junior doctors, and they use that to check. You know, if the patient might need a refraction. That’s my kind of understanding. So, I didn’t know, because, you know, earlier I was imagining everybody does refraction with a retinoscope, but we know that certainly a lot of our students and optometrists in practice more are using an auto-refractor rather than actually getting there ret out, and you know that doesn’t work too well in the paediatric population. I don’t think, or certain patients.

	They do use the auto-refactor on a lot of patients to decide whether they are going to refer them for a refraction or you know what they are going to do, or whether they think something else can be done. I certainly notice that you know a lot of times that’s mentioned in the records.

	To use it in place of something like retinoscopy. And, as xxxxx has said, in a paediatrics. Using automated tests and things like that. They are not the best at all the kids won’t sit still there’s a lot of off axis errors. You’ll get really weird results. I think that’s when the skill of the refractor or the refractionist, if you like, comes into it. And there is no substitute at all for having a retinoscope for that purpose.

	Just to add to the complexities of that question: a cyclo auto-refraction. Where does that sit in that decision-making. I would say it would create even more off axis errors and really weird and wonderful things. Now, speaking from the point of view of someone who doesn’t use an auto refractor, I get my hands dirty when I do my refractions- I’m old school. I use a ret a trial frame, and if I do is subjective, I will use a trial frame. I don’t use phoropters or anything like that.

**Cultural changes- Some optical practices refuse eye care to younger children**.	And a lot of places a lot of practices just turn children away. Sorry we don’t do, children.

	Because you’ve got an NHS contract, and that contract says that you will see everyone and you don’t just get to say, oh, I’m only going to see them when they read.

	When I was looking for a pre-reg post I purposefully had to make an effort to find somewhere that will actually refract children under five. Lots of places just say we don’t do children.

	I think that there’s this sort of financial aspect to this. So, in your community optometrists, they’ve having a child under five is not particularly cost-effective. The time it takes to dilate, maybe bring them back, etc. And the time it takes to actually refract and all that. Yeah, and whether it’s cost-effective.

**Dynamic retinoscopy**.	It kind of struck me that refraction you know. You do it with a retinoscope, but you also use the retinoscope to do dynamic retinoscopy as well and I’m not kind of sure if that’s taught to people.

	But the issue comes when the way our clinics is to set up is often the children are given cyclopentolate to put in at home, and so that comes to me cyclopleged. So, if I need to do dynamic ret then that on another day so I need to bring them back some other time.

	I don’t know what their accommodative functions are like so the kind of patient care would improve definitely if retinoscopy were used as a tool as a cover test as used as a tool or motility, or something like that.

	In a hospital setting, I would, I would do it. If a child is coming in with reading and difficulties. I think it’s a really good way to, you know quickly assess their cognitive function, and to see how much lag like you said how much of the prescription is significant to them.

	Yes, we do dynamic ret, it’s a bit hit and miss it. It’s not done on all patients, because some patients come in already dilated. So, on the un-dilated ones I would do it, in the VPD (visual perception difficulties) patients and Down’s syndrome children, we would do it in as well.

	All I was going to say is when I trained as an orthoptist, I had never been told about dynamic retinoscopy at all. I didn’t know it existed. And then, certainly, when I did my optometry training recently, that. XXXX put it through to us, and I thought it was amazing like, wow, what is this? yeah, just didn’t know about it.

	The orthoptists prefer that I kind of supervise or talk about it, because they don’t necessarily feel comfortable.

	To just say the optometrists on the whole, aren’t confident doing dynamic retinoscopy. But your average orthoptist would not be either. So, yeah, I think it’s this dark art for a lot of people.

	With the accommodative problems dynamic retinoscopy trying to explain that to my optom colleagues is, it’s very difficult. It’s not well understood.

	It’s always struck me as bonkers that any accommodative defects come through to an orthoptic department. But then, how do you assess accommodation?

	I can’t answer that, as I’m not an orthoptists I love dynamic retinoscopy it lets me know whether or not a child needs a prescription

	I was just like about to say I remember in second year having the lecture, but we never actually had that sort of practical side to it, but like we did with sort of other techniques.

	We did do it. We did learn it, and we did have to use it in clinic like practice and it was part of our optics practical exam in second year. So, we do it as part of the exam. But I haven’t really used it on patients at all, so I would say, if somebody gave it to me now, it’d probably be back to square one.

	Yes, at a very basic level. Nothing that would be confident to go and do it.

**More cost-effective**.	We couldn’t get any optoms out there at all. You know it’s too far out. It’s not enough money which is where the ophthalmologists would then do refraction and the fundus check.

	But it’s a shame that it still has to be okayed by a doctor, and you think you know what you’re doing. You’ve been doing it for so long. But you’re not getting that final sign-off yourself. That’s really frustrating that we can’t get ourselves across the line.

	I think it’s a waste of NHS time because you can’t book as many patients into the clinic.

	I was just really going to see the major advantage would be cost-effective.

	We couldn’t get any optoms out there at all. You know it’s too far out. It’s not enough money which is where the ophthalmologists would then do refraction and the fundus check.

**Patients would prefer one appointment**.	I mean could the advantage you’re asking advantages. Could it be that the patients have to make less visits to the clinic.


Note: When participants mention “BV,” they are referring to binocular vision; “ret” refers to either retinoscope or retinoscopy; “DO” refers to dispensing optician; “paeds” refers to paediatrics; “optoms” refers to optometrists and “cyclo” refers to cycloplegic refraction.

## Discussion

A significant number of orthoptists across the UK are already conducting refractions on behalf of ophthalmologists. One likely contributing factor is the challenge in recruiting optometrists to work in paediatric refraction clinics, as they often enjoy roles in community optical practices or NHS clinics where they manage ocular pathology (Miller, 2023). Another reason is the difficulty in justifying paediatric ophthalmologists carrying out refractions, especially considering the chronic shortage of ophthalmologists across the UK. According to a Royal College of Ophthalmologists Workforce Census, 67% of hospital eye units are utilising locum doctors to fill consultant posts, representing a 52% increase since, 2016 (RCO, 2019).

Although orthoptists in the UK are authorised to perform refractions, the optical prescriptions that result from their refractions must be signed by an ophthalmologist or an optometrist irrespective of the number of years of refraction experience of the orthoptists. This limitation persists despite participants recognising that the final prescription often stems from decisions made by a multidisciplinary team. In contrast, orthoptists practising in Australia have diverse roles, including paediatric triage and refraction (Queensland Legislation, 2024).^1^ Orthoptists can prescribe spectacles in the course of carrying out duties at a public health facility; or under the supervision of an optometrist or medical practitioner; or to a person who has had an ocular health examination conducted by an optometrist or medical practitioner within the 12 months prior the referral (Queensland Legislation, 2024).^2^ A major tertiary hospital in Australia utilised orthoptist-led triage clinic for non-urgent ocular motility or vision disorder referrals, to manage increased demand, expedite care safely, and refer appropriate cases to community centres (Scheetz *et al*., 2016). These authors concluded that orthoptists possess the requisite skills to offer comprehensive care to children referred for ocular motility and/or vision-related disorders, as there was close alignment between orthoptists and medical practitioners in performing comprehensive eye examinations (Scheetz *et al*., 2016). In France, orthoptists typically work under ophthalmologist supervision and have a scope of practice analogous to their UK counterparts (Thomas *et al*., 2011). Since 2023, individuals aged 16–42, free from contraindicated conditions, have the option to voluntarily undergo eye examinations conducted by orthoptists (Legifrance.gouv.fr, 2007).^3^ These examinations aim to provide updated prescriptions for spectacles and contact lenses to address conditions such as myopia and astigmatism (Legifrance.gouv.fr, 2007).

In the present research, participants in both groups unanimously agreed that orthoptists wish to conduct refractions and issue optical prescriptions exclusively within NHS settings whilst all patients are under the supervision of an ophthalmologist. Several reasons were cited for this preference, with the paramount concern being patient safety, particularly in relation to pathology detection. In Italy, the optometric system primarily revolves around optometrists conducting refractions without the requirement to identify ocular pathology (Cheloni *et al*., 2021). A study conducted by Cheloni *et al*. (2021) revealed that in Italy, as many as 19% of patients might have asymptomatic pathologies which remain undetected during the optometrist’s examination (Cheloni *et al*., 2021).

Concerning training, some participants agreed that contemporary student orthoptists receive more refraction training in their undergraduate degree than they would have historically. Clinicians also concurred that substantially more post-graduate training would be necessary for orthoptists to proficiently conduct refractions. The need for this is supported by the observation that participant orthoptists who are already undertaking refractions typically described attending post-qualification training courses on this topic to help them develop the necessary skills. This perspective is supported by research findings, which demonstrate a positive correlation between increased hours of practice and enhanced performance in retinoscopy (Estay *et al*., 2023). Some comments highlighted that orthoptists undergo comparable training in refraction to that of ophthalmologists however, the ability to issue optical prescriptions lies solely with the ophthalmologists and optometrists. This fact has been confirmed through a collaboration between BIOS and the Royal College of Ophthalmologists, which disclosed that the training offered to orthoptists in refraction closely aligns with the knowledge and skills on this topic imparted to trainee ophthalmologists (BIOS, 2024).

Another prevalent theme emerging from the focus groups was that optometrists and ophthalmologists are increasingly lacking in the necessary skills and/or experience for conducting sight tests of young children and patients with special education needs (SEN). This observation is substantiated by research indicating that deficiencies in knowledge, skills, and confidence act as barriers to paediatric eye care, consequently reducing the likelihood of optometrists examining young children (Wilson *et al*., 2023). The focus groups also discussed the issue that patients with SEN and younger children, particularly those under the age of five, are sometimes denied eye care in community optical practices. This finding is corroborated by the literature repeatedly showing that young infants and individuals with SEN regularly encounter difficulties in accessing eye care (Shah, Evans and Edgar, 2007; Wilson *et al*., 2021).

Both focus groups indicated that due to the operational procedures in most NHS HES clinics, there is a significant chance that many accommodative anomalies in pre-school children are largely undetected. This is because in the HES cycloplegic drops are typically administered before an optometrist sees the patient, so the optometrist cannot assess accommodative function with dynamic retinoscopy. Orthoptists seldom practise retinoscopy during their clinical practice as they are prohibited from prescribing. This in turn results in a decline in their retinoscopy skills including dynamic retinoscopy. Both factors mean that accommodative anomalies are potentially undiagnosed in the younger children when subjective responses are not possible. Participants felt that the ability to refract and manipulate prescriptions aids clinical decision-making leading to better management. For example, there was commentary to suggest that it would be useful to be allowed to legally adapt an existing recent prescription, in order to manage a number of binocular vision anomalies without the need for an additional eye test.

Cost is another potential barrier to the patient, their parent/carer and the NHS. Hospital eye service (HES) treatment for eye conditions frequently necessitates regular absences for children from education and carers from employment, which are commonly cited reasons for non-attendance at eye clinics. Implementing dual orthoptic and refraction appointments with an HES orthoptist for vision testing could prove beneficial for both parents and children, as it addresses known barriers to eye care such as costs and time (Donaldson, Subramanian and Conway, 2018). Furthermore, by minimising the number of appointments and the number of full sight tests needed, the NHS would also benefit from the cost and time saving and reduced administrative expenses.

## Conclusion

Overall, there were many potential advantages foreseen in relation to permitting orthoptists to issue optical prescriptions as part of their work exclusively within the HES. Disadvantages could be minimised or eliminated by restricting these refractions to limited circumstances with appropriate training and safeguarding procedures. In the HES, roles are increasingly defined by competencies rather than professional titles. Each team member is accountable for working within their area of competence, while leaders are responsible for ensuring proper training and continuous oversight. A legal change allowing orthoptists to issue optical prescriptions for young children under their care in the HES would represent a further development in the shift towards competency-based roles.

## Footnotes

https://www.legislation.qld.gov.au/view/pdf/inforce/current/act-2009-hprnlq.

https://www.legislation.qld.gov.au/view/pdf/inforce/current/act-2009-hprnlq.

Decree No. 2007-1671 of 27 November 2007 establishing the list of acts that may be performed by orthoptists and amending the Public Health Code (regulatory provisions) – Légifrance (legifrance.gouv.fr).
